# Factors predisposing women and children to indoor air pollution in rural villages, Western Kenya

**DOI:** 10.1186/s13690-022-00791-9

**Published:** 2022-01-29

**Authors:** Gabriel O. Dida, Patrick O. Lutta, Paul O. Abuom, Tomislav Mestrovic, Douglas N. Anyona

**Affiliations:** 1 Department of Health Systems Management and Public Health, Technical University of Kenya, Nairobi, Kenya; 2 School of Public Health and Community Development, Maseno University, Kisumu, Kenya; 3 Deparment of Environmental Science, Maseno University, Kisumu, Kenya; 4 University Centre Varazdin, University North, Varazdin, Croatia; 5 Institute for Health Metrics and Evaluation and the Department of Health Metrics Sciences, University of Washington, Seattle, USA

**Keywords:** Indoor air pollution, Rural villages, Kenya, Biomass fuel, Acute respiratory infection

## Abstract

**Background:**

Indoor air pollution (IAP) remains a major global public health hazard more so in developing countries where use of fossil fuels is still very common. However, despite the popularity of kerosene and fuelwood as energy sources among many households in the Sub-Saharan Africa, little is known about their health effects and the predisposing factors particularly on those with direct exposure. This study sought to relate indoor air pollution exposure to self-reported prevalence of respiratory outcomes including (sputum production, congestion, breathing difficulties, eye problems, fatigue, and headaches and wheezing) among women and children of Trans Nzoia County, in the rural villages of western Kenya.

**Methods:**

In this cross-sectional survey, simple random technique was used to select 251 households from 14 villages. Households were the sampling units, while the woman of the household with/or in custody of a child aged less than 5 years old were the unit of analysis. A total of 251 women with/or in custody of a child aged less than 5 years old took part in the study. A structured questionnaire was used to collect information on cause and effects of IAP among women and children. Data was analyzed descriptively and inferentially. We used Poisson generalized linear models with IAP symptoms and indoor cooking as dependent variables and household profiles and other socio-demographics as independent variables to identify the factors that affect health outcome.

**Results:**

Mean age of respondents was 36.49 years, (95% CI [35.5, 37.5]). Most (64.5%) houses were semi-permanent, with 58.6% having an average kitchen size (5.6 m^2^). Wood and kerosene were the most preferred fuel types for cooking (96.8%) and lighting (97.4%), respectively. Smoke from the wood was identified as the dominant (96.8%) source of IAP. Most women (92.0%) and children (95.4%) had coughs of varying intensities during the year, while 31.5% of the women reported wheezing. About 98% of them experienced fatigued and headaches. Use of wood fuel was associated with increased coughing (*p* = 0.03), phlegm (*p* = 0.02), wheezing (*p* = 0.04), eye problems (*p* = 0.03) and headaches (*p* = 0.01) among women and children in the previous 24 h. Education level, ventilation, main fuel source used in 24 h, indoor cooking and house type were significantly associated with IAP health effects (*p* ≤ 0.05).

**Conclusions:**

Supporting the impoverished households and increasing their level of awareness on health-effects of IAP occasioned by use of biomass fuel while cooking indoors may be the first step in implementing a programme aimed at reducing exposure among rural households in Trans Nzoia County, in rural parts of Western Kenya.

## Background

The World Health Organization recently ranked Indoor Air Pollution (IAP) from solid fuels among the top risk factors responsible for preventable loss of disability-adjusted life years [1, 2]. Indoor air pollution causes an estimated four million deaths annually across the world with an overall attributable mortality rate among women being about 50% higher than that of men [3–5]. Up to 70% of approximately 1.3 billion people living in poverty are women, many of whom live in female-headed households in rural areas [6, 7]. Sadly, most of these women are exposed to high smoke levels for prolonged periods lasting more than 5 h daily during food preparation [8].

Indoor air pollution is a serious health hazards and is listed as the leading environmental risk factor for female mortality, accounting for 5% of all female deaths in the developing world — even more than those caused by malaria each year [8, 9]. The International Energy Agency (IEA) [10] predicts that by 2030, 1 billion people will still be lacking access to electricity while 2.6 billion people will not have access to clean cooking fuel. Currently, an estimated 2.5 billion people worldwide rely on biomass fuel including dung, wood, agricultural residues and coal for cooking, heating and lighting [11, 12]. In Africa, over half a billion people (representing 78% of the population) still rely on biomass fuel for cooking and heating [13]. In Kenya alone, more than 68% of the population relies on biomass fuel for cooking and heating with up to 95% of the energy consumed in rural areas being in the form of wood, agricultural residue and animal waste [14]. Reliance on biomass fuel for cooking by up to 84% of the Kenyan population has also been reported in other studies (REN21 [15].

When used in simple cooking stoves, biomass fuels emit substantial amounts of toxic pollutants presenting a major health concern. In households with limited ventilation (as is common for many households in rural areas), exposure levels for household members, particularly women and young children who spend a substantial duration indoors is quite high and well above the World Health Organization (WHO) guidelines and national standards [5, 16, 17]. Indoor air pollution (IAP) from domestic biomass combustion is therefore a serious health concern that disproportionately affect women and young children in rural areas of low-income countries.

In Kenya, low-income households rely quite heavily on biomass fuel for cooking and heating, resulting in high levels of particulate matter and carbon monoxide (CO) emission in the houses. In some instances, unacceptably high emission levels of up to 1000 times higher than the acceptable limit by the Environmental Protection Agency (EPA) – PM_10_, which limited PM_10_ concentrations to 50 μg/m^3^ based on an annual average, and 150 μg/m^3^ based on a 24-h average [7, 14, 18]. In many households, most of the cooking is done by women (main cooks), often in poorly ventilated cooking spaces often characterized by heavy deposits of soot on the inside walls and roofs [19, 20].

During cooking, it is common practice to find the women and their young children together in the kitchen; younger ones often strapped on their backs. Prolonged stay in such environments exposes the women and their children to respirable suspended particles (RSP) resulting from incomplete combustion of biomass fuel. This confines women and young children at a particularly higher risk of developing acute respiratory infection (ARI) owing to the prolonged duration of time spent in the kitchen compared to men [21]. Moreover, children are at a higher risk due to their low immunity status [22].

Despite IAP resulting from smoke being a serious cause of death among children in developing countries, efforts to tackle childhood ARI are still minimal and more often fall short of target. Evidence from previous studies indicate that despite increased knowledge of ARI and its causes especially among communities where interventions have been conducted, change in behavior and practice is yet to be realized [21, 23]. This calls for specific actions focused at preventing further deaths from indoor air pollution [2, 24]. Cutting down on IAP is the first step to better respiratory health among women and children and an effective way of reducing associated health risks. Credible empirical evidence on the extent of IAP in rural areas of developing counties is, therefore, a pivotal step for rapid intervention.

In a relatively cold region such as Trans Nzoia County in Kenya [25, 26], residents have a habit of closing windows and doors most of the time even when using firewood as a source of fuel for cooking and warming themselves. Such acts are likely to expose the household occupants to IAP, more so the women and under five-year-old children who are often closest to the cooking stove. House structure, occupants and sources of fuels may pose unseen health morbidity and silent mortality unknown to policy makers. This study thus sought to establish the factors predisposing women and children to IAP and their health outcomes in the rural Trans Nzoia County, located in western parts of Kenya.

## Methods

### Study setting and design

This study was conducted in a rural setting within Kaplamai location of Trans-Nzoia County in the western part of Kenya. The location was purposely selected based on its’ unique transcultural inhabitants with unspecified or mixed characteristics and indefinite boundaries. The location receives moderate to heavy rainfall throughout the year with a peak between March and May. The main cash crop is maize; though subsistence farming of other food crops is practiced, alongside small scale livestock rearing. Kaplamai location has 2 sub locations and 14 villages with an approximate population of 25,000 [27, 28]. The major source of energy for cooking, lighting and heating in the area are based on solid fuels, including fuel wood, charcoal, paraffin, crop chaffs and animal cake with fuel wood expected to contribute to a larger share in total energy supply in the region because of easy excess to forests.

In this cross-sectional survey, households were the sampling units and the woman of the household with/or in custody of a child aged less than 5 years old were the units of analysis. Households with a smoker were excluded from the study. We randomly selected 300 respondents from which 49 households were dropped during the analysis, as they did not meet the inclusion criteria. Out of the 49 households dropped, 16 households had no children < 5, 15 had respondents below the age of 18, while 18 had at least one person who smoked. A structured questionnaire was used to collect data from the 251 selected respondents.

### Variables of interest

#### Dependent variables

The dependent variables studied included symptoms of IAP (which were history of child and mother coughing for weeks or months, recurring headaches, child and mother wheezing, sore throat, nasal congestion, breathing difficulties, eye problems and fatigue) and indoor cooking practices. Relationship between of each of the dependent variable was measured in relation to the independent variable of primary interest. Occurrence of presumptive respiratory systems were self-reported by the respondents and here, we asked respondents to report on an episode of presumptive IAP symptoms the previous night or frequency of occurrence of the same within the past 6 months or one year from the time of data collection. In the IAP symptoms questionnaire, respondents were asked to state their experience with the symptoms that they related with IAP. We also tested the women’s knowledge on the possible intervention methods.

#### Independent variables

The independent variables of interest included: demographic and socio-economic factors, as well as predisposing factors influencing the frequency of respiratory health symptoms on exposed women and the children. The potential influences of socio-demographic variables (education, age, number of individuals per household, kitchen location and size, presence of open eaves or windows, marital status, indoor cooking behavior, main fuel source, main fuel used for cooking and lighting in the last 24 h) on respiratory outcomes were assessed in the analyses.

#### Measurements

Age was measured in terms of number of complete years and was analyzed as a continuous ratio variable. Education was taken as an ordinal variable and measured by the level of education attained by the respondent (no formal education, primary level, secondary level, tertiary level and university level). Marital status was taken as a multinomial variable and measured as being either married, single, widowed, separated or divorced. The number of children per household was taken as a discrete ratio variable and measured by the number of children given. Location of the kitchen was taken as a multinomial variable and measured by the location of the cooking spot (within main house, in a separate house, in a separate room within the main house. The size of the kitchen was taken as a multinomial variable and measured by the kitchen size categories (small, medium or large). Presence of eave space or window were taken as nominal binary variables and measured as either being present or absent. Similarly, indoor cooking behavior was taken as a binary variable and measured as either indoors or outdoors. The main fuel used for lighting or cooking was taken as a multinomial categorical variable and measured by the type of fuel cited (electricity, kerosene, solar, firewood, LPG gas, charcoal, plant residues etc).

#### Data analysis

The quantitative data generated was analyzed descriptively to summarize the data as well as inferentially. Chi-square and correlation test were used to determine the statistical significance of key observations and differences seen in cross-tabulated variables. Since our dependent variables were counts of symptoms (i.e. frequency of respiratory symptoms encountered by women and their children), we tested the normality of the dataset to decide whether the mean was applicable as representative value of the data or not. We concluded that the normality assumption of Ordinary Least Squares (OLS) regression was inappropriate and opted to use Generalized Linear Model (GLM). We also assessed for the absence of multicollinearity. Universally, the well-known GLMs for count variable are Poisson or a negative binomial distribution. Although both Poisson and negative binomial regression models are specifically designed to analyse count data, they differ in principle of assumptions. Poisson regression model, which assumes that distribution of mean and variance, are equal, best fitted the assumption and was used in this case. We used number of respiratory health symptoms (sore throat, cough, congestion, wheezing, breathing difficulties, eye problems, headache and fatigue) faced by women in this scenario as dependent variable in a Poisson regression model as highlighted below;

i $$ Prob\ \left({Y}_i=\raisebox{1ex}{${y}_i$}\!\left/ \!\raisebox{-1ex}{${x}_i$}\right.\right)=\frac{e^{-{\lambda}_i}\ {\lambda}_i^{y_i}}{y_i!} $$

Where, *yi* represents the number of respiratory symptoms faced by the women in i^th^ households and varies across households (i = 1, … ., n). Poisson distribution was assumed to have conditional mean (*λ*_*i*_), which in turn depends on vector (*x*_*i*_) of exogenous variables. The most appropriate functional form of *λ*_*i*_ as applied is loglinear model expressed as:

ii $$ \ln {\lambda}_i={\beta}_i{x}_i+{\epsilon}_i $$

Where, *β*_*i*_ is a vector of coefficients, *x*_*i*_ is a vector of explanatory variables and *ϵ*_*i*_ represents unobservable family specific random effect that affects woman and child’s health status in the house. The explanatory variables included the common symptoms of IAP, dummy for fuel source, dummy for kitchen type, number of individuals in house, education status, employment, presence of open eaves, indoor cooking, main fuel source for cooking and lighting (measured as proxy with the number of females involved in cooking) were expected to impact on respiratory health symptoms. The reference fuel used in the multiple comparison was wood fuel because of its widespread use in most households (96.8%) compared to other fuel types within the study setting. The reference housing structure was a semi-permanent house while the average kitchen size (60 sq. ft) detached from the main house was used as the reference kitchen type. Cross ventilation type was used as a reference to other ventilation (through ventilation and door only ventilation) types. Dummy for IAP symptoms (child or mother cough =1, if not = 0), (child or mother wheezing =1, if not = zero) and indoor cooking status (Indoor = 1, if not = 0). The same dummy classifications were applied to other variables that required such classifications.

In relating background factors to IAP, we employed a generalized linear model (GLM) to investigate the predisposing factors to IAP using R 2018 software (R Core Team, 2018. R: https://www.R-project.org/). The independent variables were the demographic and socio-economic factors (education, total number of members, sources of family income, marital status, house size, availability of windows and eave status) along with other predisposing factors such as indoor cooking behavior, preferred fuel for lighting and main fuel sources. The response variable were the cumulative number of respiratory health symptoms. The choice between Poisson and negative binomial regression of the GLM depended on the nature of the distribution of the dependent variable. The Deviance and Pearson Chi-Square Goodness of fit statistics indicated the presence of over dispersion and thus we accepted the null hypothesis of α = 0, rejecting the negative binomial regression in favour of Poisson regression model. The final model was built from that premise and then simplified by omitting those predictors that contributed little to the original model using Akaike Information Criteria (AIC).

### Ethical considerations

Participation in this study was voluntary for all participants and therefore written and verbal consent was sought and obtained from the respondents prior to data collection. Ethical approval to conduct the study was also obtained from Maseno University Ethical Review Committee. The provincial administration was briefed about the study and its’ objectives, procedures and the overall requirements. Thereafter, permission was sought and obtained from the Trans Nzoia County Commissioner for the study to be conducted in their area of jurisdiction.

## Results

### Socio-demographic characteristics

A total of 251 women respondents took part in the study. The mean age of study respondents was 36.49 ± 10.86 years (± SD), with most (65.9%; 95% CI: 60.3–71.0) of the respondents being between 18 and 37 years old. Most (86.2%; 95% CI: 70.8–82.3) respondents were married, with 8.4% (95% CI: 4.2–8.9) being single and 5.2% being widowed. Most (46.2%; 95% CI: 38.7–51.5) households had 2 children aged below five years, and another 42% (95% CI: 38.1–57.0) had 3 children below 5 years old. Slightly over half 127 (50.6%; 95% CI: 47.0–61.5) the respondents had attained primary school level of education, 77 (30.7%; 95% CI: 28.7–32.2) had attained secondary level, 10 (4.0%; 95% CI: 1.1–4.8) had attained tertiary level of education, while 37 (14.7, 95% CI: 11.9–16.3) did not have formal education. The main source of income for most 164 (65.3, 95% CI: 56.2–72.7) households was farming, while other sources included formal employment for 45 (17.9, 95% CI: 16.4–18.3) households, business for 39 (15.5, 95% CI: 14.5–16.2) households, and other unspecified means for 3 (1.2%) households. Most (55.3%) households made between Ksh. 5000 and 8000 as household income monthly.

### House and kitchen characteristics

Most 162 (64.5, 95% CI: 56.3–69.5) respondents lived in semi-permanent housing structures, 57 (22.7%; 95% CI: 18.2–24.7) in temporary structures, and 32 (12.8%; CI: 9.3–14.1) in permanent houses. More than half 125 (49.8%; 95% CI: 45.4–52.6) of the houses were in good condition (Table 1). Most 147 (58.6%; 95% CI: 50.1–61.4) respondents had average sized (60 sq. ft) kitchens, 82 (32.7%; 95% CI: 29.1–33.8) had small sized (< 60 sq. ft) kitchens while 22 (8.8%; 95% CI: 7.4–.9.3) had large sized kitchens (> 60 sq. ft). Most (212, 84.5%; 95% CI: 77.6–.90.0) of the kitchens were located in a separate building from the main house and were enclosed. Cross ventilation was the most common (110, 43.8%; 95% CI: 39.8–44.1) type of ventilation used in cooking places, with 78 (31.1%; 95% CI: 28.2–33.3) and 63 (25.1%; CI: 24.5–26.3) of the cooking places having through and door only ventilations, respectively. Most 159 (63.3%; 95% CI: 59.6–64.2) cooking places lacked eave spaces, with only a small proportion (12.4%; 95% CI: 12.0–12.8) of the cooking places being well lit.

**Table 1:** House and kitchen characteristics

Interest	Observation	N	Percentage (95% CI)
**House type and kitchen condition**			
Main house type	Temporary	57	22.7 (18.2–24.7)
	Semi-permanent	162	64.5 (56.3–69.5)
	Permanent	32	12.7 (9.3–14.1)
General condition of house	Excellent	4	–
	Good	125	49.8 (45.4–52.6)
	Fair	112	44.6 (38.2–49.1)
	Poor	10	4.0 (0.1–4.8)
**Kitchen size, location and state**			
Kitchen size	Large	22	8.8 (7.4–.9.3)
	Average	147	58.6 (50.1–61.4)
	Small	82	32.7 (29.1–33.8)
Location of kitchen	Separate building	212	84.5 (77.6–.90.0)
	Separate room within main house	34	13.5 (10.1–14.0)
	Main living area in house	5	–
State of the kitchen	Enclosed	210	83.7 (78.2–88.8)
	Semi closed	41	16.3 (16.8–17.0)
**Ventilation type, presence and size of eaves and lighting of kitchen**			
Type of ventilation of cooking place	Cross ventilation	110	43.8 (39.8–44.1)
	Through ventilation	63	25.1 (24.5–26.3)
	Through door only	78	31.1 (28.2–33.3)
Presence of eaves	Yes	92	36.7 (33.7–38.4)
	No	159	63.3 (59.6–64.2)
Size of the eaves	None	159	63.3 (58.4–67.7)
	6 inches	73	29.1 (28.7–30.1)
	6–9 inches	15	6.0 (5.9–6.8)
	9–12 inches	1	–
	More than 12 inches	3	–
Lighting of cooking place	Bright and airy	31	12.4 (11.8–13.0)
	Average	169	67.3 (61.5–71.2)
	Dark and enclosed	51	20.3 (19.1–21.4)

### Household fuel characteristics

Various sources of indoor air pollution were identified within the house. The majority of households 243 (96.8%) relied on wood as their main source of fuel for cooking and heating with an equally high proportion (247, 98.4%) of respondents citing fire place as the major source of indoor air pollution. Kerosene was the most 245 (97.6%) preferred household fuel for lighting, while electricity was used by only 0.8% of the respondents (Table 2).

**Table 2:** Fuel characteristics and source of in-door pollution

Variable of Interest	Reported	N	Percentage
Main fuel used for cooking in the household	Wood	243	96.8
	Charcoal	1	0.4
	Residues	6	2.4
	Other	1	0.4
Source of fuel used for cooking in the household	Bought	84	33.5
	Gathered	167	66.5
Main fuel used for lighting in the household	Electricity	2	0.8
	Kerosene	245	97.6
	Other	4	1.6
Source of fuel used for lighting in the household	Yes	250	99.6
	No	1	0.4
Source of indoor air pollution	Fire place	247	98.4
	Kerosene lamps	1	0.4
	Other	3	1.2

### Health implications of IAP to women and children

#### Coughing among women and children under five years associated with IAP

Most women (231, 92.0%), reported having coughs of varying intensities over a 12-month period – 106 (45.9%) of whom reported coughing in the same manner (i.e. coughing persistently) on most days, while 70 (30.3%) reported coughing persistently for 1 to 2 months. Almost one-third 77 (33.2%) of the women respondents reported coughing persistently in for a period of 2 years (Table 3). Most (239, 95.4%) children had a cough the previous week, with 129 (51.2%) of the coughs being associated with increased rate of breathing. Coughing in children was significantly associated with the condition of the kitchen (enclosed or not) (Exact chi = 7.11, *p* = 0.02), while children under 5 years who stayed with their mothers in enclosed kitchen were significantly more likely to have a cough than those in kitchens that were not enclosed (OR = 3.65, 95% CI [1.34, 9.95], *p* = 0.01).

**Table 3:** Effects of indoor air pollution on women and children

Variable of Interest	Response	N	Percentage
**Respiratory problems related to IAP among women**			
Whether respondent had a cough in previous 12 months	Yes	231	92.0
	No	20	8.0
Whether respondents coughed in a similar way on most days	Yes	125	54.1
	No	106	45.9
Duration in months in the past year that respondent coughed in a similar manner	> 6	50	21.6
	3–5	63	27.4
	1–2	70	30.3
	< 1	48	20.7
Number of years respondent had been coughing in a similar manner	1	73	31.5
	2	77	33.2
	3	24	10.6
	4	4	1.7
	5	31	13.3
	> 5	22	9.7
**Respiratory problems among children related to indoor air pollution**			
Number of children under five in the household	1	111	44.2
	2	112	44.6
	3	22	8.8
	> 3	6	2.4
If children had a cough the previous week	No	12	4.6
	Yes	239	95.4
Condition of breathing for those who had a cough	Normal	122	48.8
	Faster	129	51.2
Other respiratory health problems experienced by under 5 yr olds during time of study	None	37	14.8
	Colds	206	82.0
	Others	8	3.2
**Headaches and their causes among women**			
Headache in the previous 12 months	No	5	2.0
	Yes	246	98.0
How often headaches were experienced	Every day	5	2.0
	Most days	85	34.6
	Once per week	69	28.0
	Less often	87	35.4
Factors attributed to the headaches	Smoke	12	4.9
	Colds	104	42.3
	Other	130	52.8

#### Chest problems (wheezing, congestion) and sputum production among women due to IAP

Some of the respondents (79, 31.5%) reported wheezing, with a majority (63, 79.8%) of them reportedly wheezing when they had a cold. Most (28, 35%) of those who reported wheezing, had produced the wheezing sound coupled with chest congestion over the previous two years, 19 (24.4%) during the previous year, and 17 (21.2%) during the previous 5 years. Most (157, 62.5%) respondents produced sputum, with 55 (35%) of them experiencing it on most days. Most (56, 35.5%) of those who produced sputum reported producing the sputum in a similar fashion for more than 5 months, while 52 (33.6%) reported producing it for a period of 2 years prior to the study. Majority (over 70%) of whom cooked in houses with poor ventilation and of smaller size. Sputum production was significantly associated with house type (Exact chi = 7.29, *p* = 0.03).

#### Eye infections and headaches among women resulting from IAP

Most (233, 92.8%) women respondents experienced various eye problems. Over 87% had watery eyes, 167 (72.1%) had eye irritation, 101 (43.3%) had red eyes, 61 (26.6%) had sore eyes, while 27 (11.2%) cited other eye problems. Majority of the women (246, 98.0%) experienced headaches within the past 12 months, with a large proportion of those (85, 34.6%) reported experiencing the headaches on most days, 28.0% once a week, 2% every day and 35.4% less often. Only 12 (4.9%) of the women attributed the headaches to smoke, while 42.3% attributed it to cold.

#### Relating background factors to IAP

**Fig. 1:**
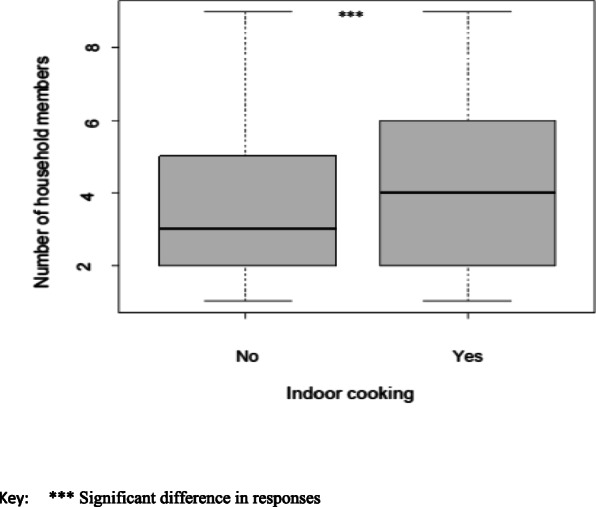
Box plot indicating association between household members and indoor cooking behavior

**Fig. 2:**
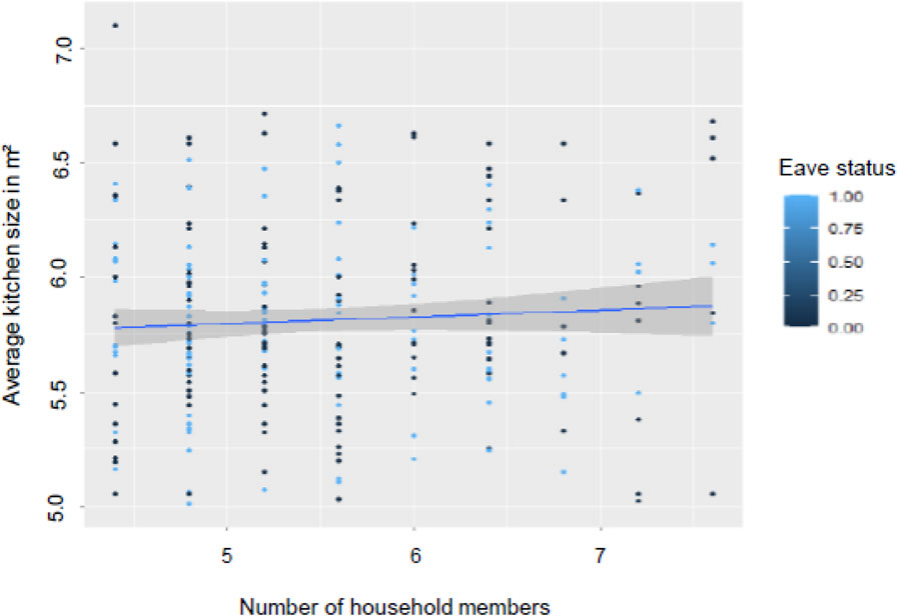
: Multi-correlation results indicating the relationship between number of individuals, indoor cooking behavior and kitchen eave status

In the estimated Poisson, factors on intergenerational relationship were highly significant as shown in Table 4. The ventilation status coefficient variable showed a negative and significant relationship suggesting that as cross ventilation or ventilation of any sort increased, the IAP symptoms tended to decrease. Likewise, a negative association between household size and indoor air pollution was observed. This was the case with house size as well. The results demonstrated that indoor cooking and main fuel source used have significant positive impact on frequency of health symptoms. Empirical analysis further showed that there was significant association between household members and indoor cooking behavior (*p* = 0.0001; Fig. 1). However, no significant correlation existed between ventilation type including status of eaves and windows and number of individuals in the house in the coefficient of multiple correlation analysis (Fig. 2).

**Table 4:** Poisson generalized linear model relating explanatory variables to IAP and indoor cooking

**Relating explanatory variables to indoor air pollution symptoms**				
**Explanatory Variable**	**Estimate**	**SE**	**Z-value**	**Pr(>|z|)**
Education	0.06	0.02	3.28	< 0.001
Ventilation	−0.03	0.01	−4.07	< 0.001
Main fuel used in 24 h	0.02	0.01	3.77	< 0.001
In-door cooking	0.46	0.01	3.71	< 0.001
House type	−0.04	0.02	−2.78	0.004
**Relating explanatory variables to indoor cooking**				
**Variable**	**Estimate**	**SE**	**Z-value**	**Pr(>|z|)**
Occupation	0.06	0.02	3.28	0.001
Main fuel source	0.02	0.01	3.88	< 0.001
House size	−0.03	0.01	−4.07	< 0.001
Age	0.06	0.02	3.54	0.001

*SE Standard Error*

## Discussion

Indoor air pollution refers to chemical, biological and physical contamination of the air quality within and around buildings and structures that may result in adverse health effects for household inhabitants. This study sought to establish the factors, both socio-demographic and structural that predispose women and children to indoor air pollution among rural families living within Kaplamai location in Trans Nzoia County. Historically, biomass fuels (wood, charcoal, sawdust, animal dung, and agricultural wastes) have been used for cooking, lighting and heating in developing countries. Duflo et al. [29], noted that the use of such fuels as sources of energy has constantly been about 25% since 1975. It is estimated that by 2030 over 2.7 billion people across the world will still be dependent on biomass fuels [30]. These energy sources, which are heavily relied upon by billions of people globally, have continued to raise health and environmental concerns and are now recognized as a significant source of potential health risks to exposed populations throughout the world [31]. It is of no wonder that the Lancet Commission on Pollution and Health endorses cleaner fuels at all levels of intervention [32].

In the current study, information on the age of the household head, occupation, ability to read and level of education were assessed. In addition, the marital status, main source of family income and household composition by female aged above 18 years and children aged below five years were also assessed with the aim of establishing the extent to which these factors influence household vulnerability to IAP.

In the present study, education status, poorly ventilated kitchen, house type, as well as consistent indoor cooking could predict the risk of most of the surveyed symptoms. Similar findings were reported by Juntarawijit and Juntarawijit [33], in which education level and cooking frequency, among other variables were reported to be predisposing factors for women to IAP. The researchers also noted that every 10 additional hours spent in a kitchen per week increased the risk of coughing, wheezing, sore throat and shortness of breath among women. Housing is particularly important indoor environment since most people spend their time at home, often indoors and sometime inside the kitchen when it is cold. Long-term exposure to IAP often occur in the home. Exposure to some pollutants is more likely in to occur in households with little income while the reverse is true for other pollutants.

Given that that majority of the household heads in the current study were aged between 25 and 34 years is regarded as advantageous to these families, as members in this age group are likely to be much more aware of the sources of IAP and its potential health impacts on household members. Being middle aged, this group could also be considered as mature and more likely to be financially stable compared to the younger age sets or the elderly [34]. However, the present study established that majority of the respondents did not associate the prevailing major symptoms such cough, and headache to IAP, instead they associated some of the symptoms like headaches and persistent coughs to cold and other factors like malaria, viral infections and poor hygiene; necessitating awareness creation among the women. Financial stability is regarded as a critical determinant of the choice of fuel type used by a household. It also dictates the type, size and design of the family house as well as the location, size and design of the kitchen. The type of biomass fuel used greatly influenced the levels of IAP in the present study. All these factors are however determined by household income.

In the current study, 44.6% of women respondents were not employed, while another 20.7% worked as unskilled labourers with most (40.2%) households generating an average monthly income of between Ksh. 2001 and 5000. Consistent with the findings of this study, Kimani [35] observed that a majority of women work in the informal sector, handling small-scale businesses with meager income to even sustain their families. Insufficient household income can only allow for the most basic commodities such as food for the family forcing households to settle for the cheapest fuel sources such as plant remains and firewood that are mostly free.

Studies show that when women are overly dependent on their spouses, they are unable to make important or critical decisions especially on matters relating to family expenditures [36]. Poverty and illiteracy remain the key barriers to adoption of cleaner fuels, with the slow pace of development in many developing countries a further indication that the use of biomass fuels will continue for many decades to come for many poor households. Akin to other studies, the use of biomass fuels among poor households in Kenya is likely to remain stable or even increase in the near future, as few rural families can afford alternative fuel that is higher on the energy ladder (such as liquefied petroleum gas and electricity, which are regarded as being cleaner but more expensive) [37]. Accordingly, the choice of fuel type used by a household becomes cleaner and more convenient, efficient and costly as people move up the energy ladder; a feat that can only be achieved through economic empowerment of women.

Studies show that households at the lower income levels tend to be at the bottom of the energy ladder; and often rely on fuel that is cheap and locally available but not very clean or efficient. According to the World Health Organization, over three billion people worldwide are at these lower rungs and depend on biomass fuels-crop waste, dung, wood, leaves and coal to meet their energy needs [38]. It is estimated that globally a majority of poor households rely on biomass fuels for everyday household energy needs, with most of those exposed being women, who are normally tasked with food preparation, and children under the age of five years who are most often with their mothers in the cooking area [39]. Consistent with the WHO [38] findings, the current study established that a large percentage (96.8%) of households relied on wood as the main source of fuel for cooking and heating with crop residues, charcoal and other sources being mentioned, albeit by less than 4% of the respondents. The second most preferred fuel for cooking and heating for a majority of households after wood was crop residues. These findings were also consistent with those of [40], who reported that approximately 3 billion people in the world use solid fuels. Precisely, 2.4 billion people use biomass (wood, charcoal, animal dung, crop wastes), and the remainder utilize coal for the majority of their household energy needs because these are relatively cheaper and thus available to a majority of the people.

Various studies [41, 42] have shown that solid fuels are extensively used for cooking and home heating in developing countries, especially in rural areas. Further, studies also show that in sub-Saharan Africa, wood fuel is acknowledged as the main source of energy in most rural communities [43], with an estimated daily fuel wood consumption of 500,000 tons per day in Africa. Sanders, [44] reported that the three-stone open fire commonly used in many developing countries is only about 10–15% efficient leaving most of the energy content of the fuel wasted. Poverty contributes highly to indoor air pollution as poor households are not able to afford alternative efficient fuel such as electricity or LPG gas and thus remain vulnerable to poor health. As such, it becomes a vicious cycle for members of the affected household as they remain trapped both in poverty and in poor health [45, 46].

Besides use of biomass fuel, several other factors at both community and household levels determine the extent of indoor air pollution among households. These factors include the design of the house, size of the kitchen and its location, availability and size of eave spaces, ventilation size and design among others. In the current study, a large proportion of respondents lived in semi-permanent houses, a few others in temporary houses and only a handful in permanent houses. In terms of kitchen size, over half the households had average sized kitchen, with a small proportion (8.8%) having large sized kitchens. Kitchen size is an important contributor to indoor air pollution since burning biomass fuel indoors produces large quantities of smoke, providing a perfect avenue for human exposure to smoke especially in confined space without adequate ventilation [47].

In the current study, most (84.5%) kitchens were housed in a separate building from the main house, while 13.5% of the kitchens were located in a separate room within the main house and 2.0% were located in the main living area. Consistent with the current study findings, a similar study by Oguntoke et al. [48] in Odede area of South Africa also established that 25% of the respondents located their kitchens indoors (separate room within the main house); while a larger majority had their kitchen in a separate room outside the main house; but in close proximity to the main house. In the current study, most (84.5%) respondents had enclosed kitchens as opposed to 15.5% who had semi-closed kitchens. The state of the kitchen influences the levels of indoor air pollution, with enclosed kitchens likely to confine higher levels of smoke indoors than non-enclosed kitchens.

In the current study, most (63.3%) houses did not have any eave spaces in their kitchens, though even for those that had, the eave spaces were small sized ranging between 0 and 6 in. in size. Most (43.8%) eaves were the cross ventilation type, with another 31.1% being door ventilation type. Indoor air pollution was negatively associated with ventilation and education, while semi-permanent houses and age were positively associated with IAP. One possible explanation for this could have been that an increase in education could have increased an individual’s understanding of IAP resulting in deliberate efforts to minimize its’ effects.

In the present study, some of the houses were grass-thatched and most of had small round windows while others did not have any, while most of the semi-permanent houses with corrugated iron sheets had spacious eaves. In a study on efficiency of eave spaces, Bruce et al. [40], observed a reduction in particulate levels from 2042 g/m^3^ to 766 g/m^3^ with a slight increase in the size of eave space. Other researchers have however argued that eave spaces alone cannot protect women and children from the effects of indoor air pollution since proximity to the fire place, duration of stay in the kitchen and length of exposure differ and these also play a vital role [40].

Given that most households in the current study used biomass fuel; most of which was burnt indoors on open fires or poorly functioning stoves, often with no or limited ventilation, a large number of women and young children within Kaplamai Division are therefore exposed to high levels of air pollution, every day of the year. Limited ventilation implies that free flow of smoke from the kitchen is inhibited thus exacerbating the effect of indoor air pollution. The WHO [49] acknowledges that the greatest global burden of air pollution exposure occurs not outdoors in the cities of the developed world, but indoors in poor rural communities. Poor ventilation coupled with incomplete combustion by most of the stoves used can result in substantial emissions which in the presence of inadequate ventilation and air circulation can produce very high levels of indoor pollutants [50]. Earlier studies including those of Smith et al. [51], Collings et al. [52], Martin [53] and Ellegard [54], showed that indoor concentrations of particles usually exceed set guidelines by a large margin. For instance, 24-h mean PM_10_ levels are typically in the range 300–3000 mg/m^3^ and may reach 30,000 mg/m^3^ or more during periods of cooking. The United States Environmental Protection Agency’s standards for 24-h average PM_10_ and PM_2.5_ concentrations are 150 mg/m^3^ and 65 mg/m^3^, respectively [14]. The current study however had a discrepancy as it did not measure the actual PM_10_ or PM_2.5_ concentrations and it would therefore be difficult to compare them against the USEPA standards.

An individual’s true exposure may vary with the size of the kitchen and an individual’s proximity to the stove during periods when the stove is in use. Saksena et al. [55] reported concentrations of 20,000 μg/m^3^ near the cooking stove and much lower concentrations in the rest of the kitchen and in other rooms within the house, while Ezzati and Kammen [56] reported peak concentrations greater than 50,000 μg/m^3^ in the immediate vicinity of the cooking stove. These two studies suggest that women, children and even girls who normally seat close to cooking stoves for extended periods of time are more likely to be exposed to higher levels of carbon monoxide compared to other household members. USEPA [14] estimated that the mean (24-h level) of carbon monoxide in homes using biomass fuels in developing countries range between 2 and 50 ppm, with values ranging between 10 and 500 ppm likely to be achieved during the cooking process. This is far above the 8-h average carbon monoxide standard of 9 ppm set by the USEPA [14].

### Health outcomes of indoor air pollution

A number of poor health outcomes result from the use of biomass fuel that is normally burnt in poorly ventilated kitchens. Other factors such as duration of time that women and children spend exposed to smoke and the habit of opening or closing windows and doors while cooking also exacerbate the health risk. According to Begum et al. [57], indoor air pollution has significant influence on women and children. In the current study, over 90% of women and children experienced coughs of varying intensities over a 12-month period, with 45.9% of the women reported coughing in the same manner for several days, while 30.3% coughed in the same manner for over 1 month. Coughing was significantly associated with IAP. Inhaled particles and gases may expose women to ARI such as pneumonia which is one of the causes of morbidity in Kenya [58]. It is, therefore, likely that among adults, women are particularly at higher risk of developing ARI because of the prolonged time they spend in the kitchen preparing meals for families compared to men. Children are also at a higher risk than adults because their airways are relatively narrower and more easily obstructed and their oxygen demand relative to body weight is higher resulting in relatively larger inhaled volumes [22, 59].

There is sufficient evidence linking smoke from solid fuel use to acute infection of lower respiratory tract, chronic obstructive pulmonary disease and lung cancer [60]. Shabir et al. [61] estimates that ARI’s are the single most important cause of mortality in children aged below 5 years, and they account for between 1.9 million and 2.2 million children deaths annually. In a study of 1532 female patients above 40 years and exposed to kitchen smoke over a span of 13 years, Mishra et al., [62] observed high incidence of chronic bronchitis followed by bronchial asthma, pulmonary tuberculosis and bronchiectasis. There is also evidence, mainly from Bangladesh, that exposure to coal smoke in the home markedly increases the risk of lung cancer, particularly in women [63], while there is also mounting evidence that cooking with biomass substantially increases the risk of developing active tuberculosis [64, 65]. Another set of health problems associated with indoor air pollution reported in this study include sputum production, headaches and wheezing, all of which are as a result of IAP and which were confirmed by very recent studies in the field [66].

In the current study, a number of eye problems were reported and linked to the smoke from the cooking stove. Khalequzzaman et al. [63] and Díaz et al. [64] reported that tears coming out from the eye, eye discomfort and sore eyes, redness and itching of the eye, eye irritation, muscle weakness, fatigue, sleeping problems, stomach pains, dry mouth, and blindness linked to cataracts, trachoma, and conjunctivitis are all indicators of indoor air pollution within houses. The constant and continued exposure to smoke from biomass fuel continues to cause both long and short-term health threats to members of the household – hence the need to cut down on expose to IAP.

Studies show that although reducing exposure to IAP from solid fuels can be achieved through several interventions targeting emission sources through improved energy technology, well designed houses and provision of ventilation, encouraging behavior change and time-activity budget is also a strategy that can reduce IAP. Most current studies however suggest that the main focus should be on improved (high-efficiency and low emissions) stoves and fuels, which provide more affordable options for the poor majority in developing countries as opposed to complete shift to non-solid fuels [67]. In that regard, the Clean Cooking Alliance has singled out Kenya as one of eight priority countries for clean cookstove endowment [67, 68].

However, while use of improved stoves has been proposed as being highly effective in reducing indoor air pollution, the challenge remains on the rate of uptake of this simple technology and its usage. Experts warn that if not properly executed, these improved stoves or any other interventions for that matter might not achieve the much-desired results. A cross-sectional population-based study of 353 households in Kasarani, Kenya, established that higher environmental health literacy may help improve IAP-associated health outcomes among those using solid fuel stoves [69]. Awareness campaigns on the effects of IAP and interventions for reducing the use of biomass fuels are warranted in the sub-Saharan Africa [70].

## Conclusion

In conclusion, supporting the poor households and increasing their level of awareness on health-effects of indoor air pollution occasioned by use of biomass fuel while cooking indoors may be the first step in implementing a programme aimed at reducing exposure among rural households in Trans Nzoia County and other rural areas of Kenya as well. There is also a need to redesign the kitchens to allow adequate ventilation, while cooking points should be separated from the dwelling units to reduce negative health outcomes. In addition, keeping children out of the kitchen during cooking should be encouraged to protect them from illnesses triggered by IAP. Successful implementation of improved stoves requires active participation by those directly affected (particularly women), with cooperation from other household members and the county government though related sectors like health, energy, environment, housing and planning.

## Data Availability

Datasets generated during the current study are available from the corresponding author by written request to legitimate scientific investigators capable of using the data.

## Abbreviations

ALRI: Acute lower respiratory infection
ARI: Acute respiratory infection
CO: Carbon monoxide
GoK: Government of Kenya
HH: Household
IAP: Indoor air pollution
MoH: Ministry of Health
WHO: World Health Organization

## References

[1] Ezzati M, Kammen DM (2002). Evaluating the health benefits of transitions in household energy technology in Kenya. Energy Pol.

[2] Rees N, Wickham A, Choi Y. Silent suffocation in Africa air pollution is a growing menace, affecting the poorest children the most. World Heal Organ. 2019;14 https://www.unicef.org/media/55081/file/Silent_suffocation_in_africa_air_pollution_2019.pdf. Accessed 3 July 2019.

[3] Goldemberg, J., Johansson, T. B., Reddy, A. K. N Reddy A. K.N., Williams R. H. (2004). A global clean cooking fuel initiative. Energy Sustain Dev 8:5–12, 3, 10.1016/S0973-0826(08)60462-7.

[4] Saldiva PHN, Miraglia SGEK (2004). Health effects of cookstove emissions. Energy Sustain Dev.

[5] WHO (2018). Household air pollution and health. https://www.who.int/news-room/fact-sheets/detail/household-air-pollution-and-health. Accessed 17 Oct 2019.

[6] Sparknet. (2006). Scenarios for the future. Gender Issues. Working draft www.sparknet.info (accessed 3 Nov 2006).

[7] Mwangi C (2017). An assessment of impact of poverty on female headed households in Kangemi, Kenya. A research project submitted to the institute of anthropology, gender and African studies in partial fulfilment of the requirements for the degree of master of arts in gender and development studies.

[8] Miller, G. and Mobarak, A. M. (2013). Gender differences in preferences, intra-household externalities, and low demand for improved cookstoves. National Bureau of economic research working paper. No.18964, NBER WorkingPapers, National Bureau of Economic Research, Inc. 58 pgs.

[9] Sophie B (2013). Solid fuel use for household cooking: country and regional estimates for 1980–2010. Environ Health Perspect.

[10] IEA. World energy outlook 2012. Paris: International Energy Agency (IEA); 2012. https://www.iea.org/topics/world-energy-outlook. Accessed 11 May 2019.

[11] IEA/OECD. 2009. World Energy Outlook . https://www.oecd.org/iea/. Accessed 11 May 2019.

[12] Rosenthal J, Quinn A, Grieshop AP, Pillarisetti A, Glass RI (2018). Clean cooking and the SDGs: integrated analytical approaches to guide energy interventions for health and environment goals. Energy Sustain Dev.

[13] Ekouevi K, Tuntivate V. Household energy access for cooking and heating: lessons learned and the way forward: World Bank publications; 2012. 10.1596/978-0-8213-9604-9.

[14] United States Environmental Protection Agency (USEPA) (1997). Revisions to the National Ambient air Quality Standards for particles matter. Fed Regist.

[15] REN21 (2016). Renewables 2016-global status report [Internet]. REN21 renewables. http://www.ren21.net/wp-content/uploads/2016/06/GSR_2016_Full_Report. pdf, 2016. Accessed Sept 28 2018.

[16] Bruce N, Perez-Padilla R, Albalak R (2000). Indoor air pollution in developing countries: a major environmental and public health challenge. Bull World Health Organ.

[17] Smith KR, Samet JM, Romieu I, Bruce N (2000). Indoor air pollution in developing countries and acute lower respiratory infections in children. Thorax.

[18] WHO. Air quality guidelines for Europe; second edition, World Health Organization, regional Office for Europe, European series no 91. Denmark: Copenhagen; 2000. http://www.euro.who.int/document/e71922.pdf. Accessed Jan 14 2019. 11372513

[19] WEO-2017 Special Report: Energy Access Outlook, International Energy Agency, 2017. (https://webstore.iea.org/weo-2017-special-report-energy-access-outlook). Accessed 14 Jan 2022.

[20] Person B, Loo J, Owuor M, Ogange L, Jefferds ME, Cohen AL (2012). “It is strong for my family’s health and cooks food in a way that my heart loves”: qualitative findings and implications for scaling up an improved cookstove project in rural Kenya. Int J Environ Res Public Health.

[21] Naz L, Ghimire U (2020). Assessing the prevalence trend of childhood pneumonia associated with indoor air pollution in Pakistan. Environ Sci Pollut Res Int.

[22] Tun KM, Win H, Ohnmar Z, Myint T, Myat KKS, Kyi S, Lwin TT (2005). Indoor air pollution: impact of intervention on acute respiratory infection (ARI) in under-five children. Reg Health Forum.

[23] Simoes AF, Cherian T, Chow J (2006). Acute respiratory infection children. Disease Control Priorities in Developing Countries.

[24] Addressing the Links between IndoorrepubAir Pollution. In: Household Energy and Human Health. Based on the WHO-USAID Global Consultation on the Health Impact of Indoor Air Pollution and Household Energy in Developing Countries (Meeting Report), Washington DC, 3–4 May 2000. Geneva Switzerland: World Health Organization; 2002.

[25] Githui FW (2008). Assessing the impacts of environmental change on the hydrology of the Nzoia catchment, in the Lake Victoria Basin. PhD Thesis, Department of Hydrology and Hydraulic Engineering.

[26] UNHABITAT (2016). East Africa climatic data and guidelines for bioclimatic architectural design. www.unhabitat.org. Accessed Jan 17 2018.

[27] Republic of Kenya (2005). Trans Nzoia District fact sheet.

[28] National Council for Population and Development (NCPD) [Kenya], Central Bureau of Statistics (CBS) [Office of the Vice President and Ministry of Planning and National Development], Macro International Inc (1994). Kenya demographic and health survey 1993.

[29] Duflo E, Greenstone M, Hanna R (2008). Indoor air pollution, health and economic well-being. Sapiens.

[30] OECD/IEA. (2011). Energy for all: Financing access for the poor. Available at http://www.worldenergyoutlook.org/media/weowebsite/2011/weo2011_energy_for_all.pdf (accessed 10 Apr 2012).

[31] Perez - Padilla, R., Schilmann, A. and Riojas -Rodriguez, H. (2010). Respiratory health effects of indoor air pollution. Int J Tuberculosis Lung Dis.

[32] Landrigan PJ, Fuller R, Acosta NJR, Adeyi O, Arnold R, Basu N (2018). The lancet commission on pollution and health. Lancet.

[33] Juntarawijit Y, Juntarawijit C (2019). Cooking smoke exposure and respiratory symptoms among those responsible for household cooking: A study in Phitsanulok, Thailand. Heliyon.

[34] Aldwin CM, Sutton KJ, Chiara G, Spiro A (1996). Age differences in stress, coping, and appraisal: findings from the normative aging study. J Gerontol Psychol Sci.

[35] Kimani EN (2004). Taking a gender perspective in the development process: A justification, in Muia, D. M. and Otiende, J. E. introduction to development studies for Africa.

[36] Klasen S. Does gender inequality reduce growth and development? Evidence from cross country regressions. In. Germany: University of Munich; 1999.

[37] Masera OR, Saatkamp BD, Kammen DN (2000). From linear fuel switching to multiple cooking strategies: A critique and alternative to the energy ladder model. World Dev.

[38] WHO. Interventions to reduce indoor air pollution. Geneva: World Health Organization, Department for the Protection of human environment, Programme on Indoor Air Pollution; 2010. (http://www.who.int/indoorair/interventions/en/). Accessed iMay 13 2019.

[39] Ardrey J (2020). Cookstoves and health, a view from the foot of the energy ladder: a qualitative study of a cookstove intervention in rural Malawi.

[40] Bruce N, Rehfuess E, Mehta S, Hutton G, Smith K, Jamison DT, Breman JG, Measham AR, Alleyne G, Claeson M, Evans DB, Jha P, Mills A, Musgrove P (2006). Indoor air pollution. Disease control priorities in developing countries.

[41] Naeher LP, Brauer M, Lipsett M, Zelikoff JT, Simpson CD, Koenig JQ, Smith KR (2007). Woodsmoke health effects: a review. Inhal Toxicol.

[42] Torres-Duque C, Maldonado D, Perez-Padilla R, Ezzati M, Viegi G (2008). (2008). Biomass fuels and respiratory diseases: a review of the evidence. Proc Am ThoracSoc.

[43] Madubansi, M. and Shackleton, C. M. (2006). Changes in fuelwood use and selection following electrification in the Bushbuckridge lowveld, South Africa. http://www.sciencedirect.com. Accessed June 19 2018. 10.1016/j.jenvman.2006.03.014 16930808

[44] Sanders R (2002). A strategy to ease indoor pollution: improving efficiency of wood burning stoves in Africa.

[45] Ray D, Bardhan P (1993). Labor markets, adaptive mechanisms, and nutritional status. Essays in honour of K.N. raj.

[46] Troncoso K, da Silva A, S. (2017). LPG fuel subsidies in Latin America and the use of solid fuels to cook. Energy Policy.

[47] Theuri, D. (2009). Rural energy, stove and indoor air quality: The Kenya Experience http://www.itdg.org. Accessed Feb 21 2018.

[48] Oguntoke O, Opeolu BO, Babatunde N. Indoor air pollutants and health risks among rural dwellers in Odeda area, southwestern Nigeria. Ethiop J Environ Stud Manag. 2010;3(2). 10.4314/ejesm.v3i2.59833.

[49] WHO (1997). Health and environment in sustainable development. Five years after the earth summit.

[50] McCracken JP, Smith KR (1998). Emissions and efficiency of improved wood burning cook stoves in highland Guatemala. Environ Int.

[51] Smith KR, Romieu I, Bruce N (1994). Air pollution and the energy ladder in Asian cities. Energy.

[52] Collings DA, Sithole SD, Martin KS (1990). Indoor wood smoke pollution causing lower respiratory disease in children. Trop Dr.

[53] Martin KS (1991). Indoor air pollution in developing countries. Lancet.

[54] Ellegard A (1996). Cooking fuel smoke and respiratory symptoms among women in low-income areas in Maputo. Environ Health Perspect.

[55] Saksena S, Prasad R, Pal RC, Joshi V (1992). Patterns of daily exposure to TSP and CO in the Garhwal Himalaya. Atmospheric environment. Part A. General Topics.

[56] Ezzati M, Kammen DM (2001). Indoor air pollution from biomass combustion and acute respiratory infections in Kenya: an exposure-response study. Lancet.

[57] Begum BA, Paul SK, Hossain MD, Biswas SK, Hopke PK (2009). Indoor air pollution from particulates matter emissions in different households in rural areas of Bangladesh. Build Environ.

[58] Muthumbi E, Lowe BS, Muyodi C, Getambu E, Gleeson F, Scott JAG (2017). Risk factors for community-acquired pneumonia among adults in Kenya: a case-control study. Pneumonia (Nathan).

[59] Rylance S, Nightingale R, Naunje A, Mbalume F, Jewell C, Balmes JR, Grigg J, Mortimer K (2019). Lung health and exposure to air pollution in Malawian children (CAPS): a cross-sectional study. Thorax.

[60] WHO (2008). Evaluating household energy and health interventions: a catalogue of methods.

[61] Madhi SA, Klugman KP, Jamison DT, Feachem RG, Makgoba MW (2006). Acute Respiratory Infections. Disease and Mortality in Sub-Saharan Africa.

[62] Mishra VK, Retherford R, Smith K. Biomass cooking fuels and prevalence of tuberculosis in India. Int J Infect Dis. 1999;3:119–29. 10.1016/s1201-9712(99)90032-2 10460922

[63] Khalequzzaman M, Kamijima M, Sakai K, Chowdhury N, Hamajima N, Nakajima T (2007). Indoor air pollution and its impact on children under five years old in Bangladesh. Indoor Air.

[64] Diaz E (2007). Eye discomfort, headache and back pain among Mayan Guatemalan women taking part in a randomized stove intervention trial. J Epidemiol Commun Health.

[65] Manibog FR (2008). Improved cooking stoves in developing countries: problems and opportunities. Annu Rev Energy.

[66] Rana J, Uddin J, Peltier R, Oulhote Y (2019). Associations between indoor air pollution and acute respiratory infections among under-five children in Afghanistan: do SES and sex matter?. Int J Environ Res Public Health.

[67] Clean Cooking Alliance. (2018). Global Alliance for Clean Cookstoves is Now the Clean Cooking Alliance. https://www.cleancookingalliance.org/. Accessed Jan 2020.

[68] Raufman J, Blansky D, Lounsbury DW, Mwangi EW, Lan Q, Olloquequi J, Hosgood HD (2020). Environmental health literacy and household air pollution-associated symptoms in Kenya: a cross-sectional study. Environ Health.

[69] Muindi K (2017). Air pollution in Nairobi slums: sources, levels and lay perceptions.

[70] Bickton FM, Ndeketa L, Sibande GT, Nkermahame J, Payesa C, Milanzi EB (2020). Household air pollution and under-five mortality in sub-Saharan Africa: an analysis of 14 demographic and health surveys. Environ Health Prev Med.

